# Supplementary material for: Learning curve and surgical outcome of robotic assisted colorectal surgery with ERAS program
Source: Sci Rep. 2022 Nov 29;12:20566. doi: 10.1038/s41598-022-24665-w (PMC9709162; doi:10.1038/s41598-022-24665-w)
Supplement: Supplementary file 1 — Supplementary Information 1. [file 41598_2022_24665_MOESM1_ESM.zip › LSC Raw data-Ñ[▒K/074/H_ñw╝╨░O▒Kñσ.pdf]

正式摘要(電子簽章：0000001391139773)

W102 / W102 ☐ 英文標題(注意:無英文姓名-請補病人基本檔!)

☐【諭取】憑證

臺中榮民總醫院

出院病歷摘要

轉入號： 轉出號：

[redacted]

(8)流水編號	
(9)入院日期	109/03/18 (2020) 直腸外科 W102 - 39
(10)轉科日期	
(11)出院日期	109/04/17 (2020) 住院天數計 30 日

診斷

- 入院：1.Rectal cancer
  2.Type 2 DM, poor controlled
  3.r/o TB infection
- 出院：1. Active pulmonary tuberculosis, right milddle lobe and left upper lobe s/p four combine therapy (AKURI-4) since 2020/04/07, sputum culture yield Mycobacterium tuberculosis (pending drug sensitivity)
  2. Rectal cancer, pT3N2a,stage IIIB (AJCC 8) s/p laparoscopic low anterior resection (LAR) and Loop ileostomy on 2020/3/25 with post-operation ileus and pelvic abscess s/p total parental nutrition (TPN) (2020/04/02~2020/04/15) and pus culture yield Pseudomonas Aeruginosm and Morganella Morgannii
  s/p Tazocine (2020/04/09~2020/04/16)
  3. Type 2 DM with poor control (HbA1C: 17.1% on 2020/03/17)
  4. Hyperlipidemia (Total cholesterol: 194 and LDL: 114 on 2020/03/17)
  5. Hypertension

主訴

- Poor appetite with body weight loss for 6kgs in recent one month

病史

- This 81 years old male has history of 1.s/p appendectomy 2.HTN 3. poor controlled DM 4.goiter.
  He compalined of poor appetite with body weight loss for 6kg in one month, he went to 大甲光田GI OPD firtst, where UGI scope showed gastritis. Then he was brought to our GI OPD, sigmoidoscopy up to 40cm FAV was done on 3/10, which revealed rectal ulcerative mass, 5cm from anal verge. Biopsy proved adenocarcinoma. Then he was referred to CRS OPD. This time, he was admitted for preop CCRT and preop survey.

體檢發現

-
  - General appearance :
    - chronic ill looking , conscious clear , without cardiopulmonary distress
    - Vital signs自動帶入date 體溫(BT) 脈搏(P) 呼吸(R) 血壓(BP) 身高(BH) 體重(BW) BMI
      20200318 35.6℃ 87次/分 18次/分 121/76mmHg 163.6cm 53.4kg 20.0
    - Vital signs: B.P. 121/76 mmHg , P.R. 87 /min , R.R. 18 /min , B.T. 35.6 ℃
  - HEENT :
    - Eyes: no anemic conjunctiva, no icteric sclera, normal EOM
    - ENT: no otorrhea, no rhinorrhea, no erythematous mucosa, no injected tonsil, no deformity
  - Neck: supple, freely movable, trachea midline,no deformity, no engorged jugular vein , no palpable thyroid or lymph node enlargement
  - Chest:
    - Inspection: Grossly normal, symmetric and fully expansion, no accessory muscle used, no operation scar, no spider angiomata, no gynecomastia
    - Palpation: no palpable mass or nodule, no subcutaneous emphysema, no tenderness
    - Ausculation: clear breathing sound
  - Heart:
    - Palpation: no thrill, no heave
    - Percussion: normal heart size
    - Ausculation: regular heart beat, normal S1, S2, no gallop, no murmur
  - Extremities: No deformity, full ranged of motion without limitation, no clubbing finger or nail cyanosis, no pitting edema, no ulcer or pigmentation, no obvious skin lesion, no varicose vein, no bone, joint or muscle tenderness

CRS CONDITION

- Karnofsky performance status: (100) Normal, no complaints, no evidence of disease
- PE of Abdomen:
- CEA: 19.09 ng/ml
- Imaging Studies:
- Colonoscope report: ulcerative tumor at 5cm FAV
- Figures:
  1. colon & rectum

手術日期及方法

- 手術日期及方法20200325
  1. Laparoscopic low anterior resection, side-to-end anastomosis, EndoGIA 60/.38mm, CEEA- 29mm. Bilateral doughnut: intact, air tight test: done, no air leak.

  2. High ligation (+) above the left colic artery.
  3. Cut margin: proximal 18 cm, distal 0 cm.
  5. Take down of splenic flexure: (-).
  6. A 12mm penrose drain placed in the pelvis.
  7. Loop ileostomy was done at RLQ

住院治療經過

- During CRS ward admission, pre-op survey was completed and it showed within acceptable range. The sputum AFS showed negative and Cardiac echography showed LVEF:57%. Due to very high HbA1C (17), we tried to control sugar tightly. Laparoscopic LAR + loop ileostomy was done on 2020/3/25. Prophylactic antibiotics was given. We removed N-G and he resumed water then liquid diet since day 2 post operation. However, abdominal distension was complained,so NPO was applied. He experienced vomiting for several times and on 2020/03/29 N-G decompression was applied with large amount of gastric content was noted. Due to prolonged NPO, we prescribed TPN since 2020/04/02. However persistent abdominal distension was noted, abdominal CT on 2020/04/07 revealed intestinal obstruction due to ahesion over pelvic region and infectious process over right middle lobe (RML) were impressed. At the same time,sputum culture yielded Mycobaceterium tuberculosis. CM doctor was consulted and he was transferred to negative pressure isolation room and began AKURIT-4 for pulmonary TB (RML and LUL) treatment.We kept current treatment and monitor condition and provided nutrition support. Due to drainage culture yielded Pseudomonas Aeruginosa and Morganella morganii, we prescribed iv Tazocin for intra-abdominal infection (IAI) treatment. After transfer to isolation room, no abdominal pain was noted. We kep NPO until 2020/04/13 and tried blend diet after followed KUB which showed no deteriorated. N-G tube was removed at the same day and no abdominal distension nor nausea or vomiting found. There were no discomfort was noted under anti-tuberculosis drug. However, massive ileostomy output(>2000mL daily) was noted. Sufficient fluid supplement was given and we followed up electrolyte and clostridium difficile toxin showed negative. The condition improved and we tappered TPN gradually. Followed up sputum AFS showed negative for three sets. We adjusted normorapid to OAD first. Discussed with family and patient about further diet control and sugar monitor program for DM. We added lower lipid agent due to hyperlipidemia. We arranged CM, CRS and Meta OPD follow up. We also instructed the issue of hypoglycemia and DM diet and home monitoring blood sugar.

合併症

- Nil

一般檢查

- 累積報告：

DATE	TIME	ALB	TP	BIL.T	BIL.D	ALKP	AST	ALT	LDH	CHOL	TG	HDL-C	LDL	UA	NH3	AMY
1090325	1733	2.8		0.4	0.1	48	14	10								
1090327	0901	2.8		0.68	0.19		9	6								
1090330	0946	2.7		0.44	0.14		6	3								
1090401	0928	2.4								101	129					

1090405	0905										40					
1090406	0900	3.6		0.44	0.18		6	5								
1090409	0922	3.5		0.69				5								
1090413	0852			0.37			10	7			117					
1090416	0913	3.6		0.4				11						2.9		

DATE	TIME	WBC	RBC	HGB	HCT	MCV	PLT	BLST	NEUT	BAND	SEG	LYM	MON	EOS	BAS
1090325	1120						297								
1090325	1725	12130	4.32	13.0	40.5	93.8	272		86.5			9.7	3.4	0.2	0.2
1090327	0850	13460		13.6			275		85.5			10.6	3.3	0.4	0.2
1090330	0911	7570		14.2			337		83.4			8.5	6.5	1.1	0.5
1090406	0901	10960		12.1	37.4		470		83.5			9.9	5.5	0.8	0.3
1090409	0851	10920	3.95	12.1			471		81.1			12.8	5.2	0.7	0.2
1090413	0905	12240		13.0			464		83.6			10.7	4.1	1.1	0.5
1090416	0904	11590	3.99	12.3	37.8	94.7	394		81.9			12.3	3.4	1.7	0.7

DATE	TIME	NA	K	CL	CA	BUN	CREAT	Mg	P	CRP	ESR	PCT	LIPASE	LACTATE
1090325	1120	140	4.0											
1090325	1733	139	3.6		8.0	10	0.69							
1090327	0901	136	4.1			12	0.91							
1090330	0946	135	4.2			25	0.99							
1090401	0928	131	4.0	94	7.4			1.5	1.9					
1090406	0900	134	4.3		8.1	15	0.81							
1090409	0922	134	4.3	95	8.4	21	0.93							
1090413	0852	133	4.6		8.8	20	0.87	2.7	2.8					
1090416	0913	134	4.1		8.7	25	0.94							

DATE	TIME	GLUCOSE	PAB	TRF	ZN	CU	KETONE	CK	CKMB	PT-P	PT-C	APTT-P	APTT-C	TRO-T
1090324	0641									9.6	10.1	25.9		
1090325	1733	205												

DATE	TIME	SP-GR	PH	PROT	GLUC	KETON	BILI	UROB	OB	NITR	LEU	RBC	WBC	EPCEL
1090327	2326	1.022	5.0	2+	500.0	Trace	Negative	0.2	2+	Negative	Trace	35-50	2-5	1-2

DATE	TIME	PH	BEB	SCVO2	PO2	HCO3	HGB	PCO2	TCO2
1090416	0809	7.365	0.4	76.6	42.9	26.2		46.8	27.6

- 放射線報告 :檢驗項目 **:CT ABDOMEN-SURVEY** 報告日期 **:1090407**

主 訴：
Result:
Finding:
Post IV contrast CT scan of the whole abdomen showed:
1. History of rectal cancer, s/p Lapa LAR and ileostomy.
2. Presence of some infiltrative soft tissue at surgical site and some fluid accumulation at presacral region, post operation change or local recurrence cannot be ruled out. Please correlate with clinical condition.
3. A drainage tube is seen with tip at presacral region.
4. Diffuse loop dilatation of small intestine with transitional zone at ileum over pelvic region (axi#59,cor#13), intestinal obstruction due to adhesion cannot be ruled out.
5. Presence of intraperitoneal free air, iatrogenic related or hollow organ perforation should be differentiated.
6. Mild ascites.
7. Presence of small renal cysts in bil. kidneys.
8. Enlargement of prostate gland with intravesical protrusion.
9. Some air inside urinary bladder is noted. On Foley's catheter.
10. Liver, pancreas, spleen and gall bladder are not unusual.
11. No significant retroperitoneal lymphadenopathy.
12. Bil. hydroceles.
13. Presence of tree-in-bud infiltration at RML, infectious process should be considered.
14. Compression fracture of T12 vertebral body. DJD change of T and L spine.
14. On NG tube.
15. DJD change of T and L spine.
Impression:
1. R/I intestinal obstruction due to ahesion over pelvic region.
2. Post operation for rectal cancer with some infiltrative soft tissue and fluid accumulation at surgical site and presacral region, D/Dx including post operation change and local recurrence.
3. Intraperitoneal free air, nature to be determined.
4. R/I infectious process of RML.

檢驗項目 **:CHEST PA VIEW** 報告日期 **:1090415**
主 訴：
Result:
Finding:
> The cardiac size is within normal limit.
> Atherosclerosis of aorta
> Bilateral hilar prominence
* Still presentation of the consolidation patch over left suprahilar lung field, tumor growth cannot be excluded suggest further evalation
> Increasing interstitial infiltration over bil. remnant lung field
> Degenerative change of thoracic and lumbar spine with marginal spur formation
> Compression fracture over T12, s/p vertebroplasty
> On CVC with tip over left brachiocephalic vein and on NG tube
> Elevation of left hemidiaphragm
* Gas dilatation of bowel loop with air-fluid level, bowel obstruction cannot be excluded, suggest further evaluation.
Impression:
As above description.

檢驗項目 **:ABDOMEN (KUB),SUPINE** 報告日期 **:1090413**
主 訴：
Result:
Finding:
> Gaseous distention of the bowel loops is noted, compatible with bowel obstruction. Suggest clinical correlation.
> The bilateral renal shadows are obscured by bowel gas.
> Degenerative changes with osteophytes of the thoracic and lumbar spine
> Compression fracture through T12 vertebra
> On nasogastric tube.
> On drainage tube superimposed left-sided pelvis.
> S/P enterostomy at bilateral lower abdomen
Impression:
As above description.

檢驗項目 **:CHEST PA VIEW** 報告日期 **:1090327**
主 訴：
Result:
Finding:
> The cardiac size is within normal limit.
> Atherosclerosis of aorta
> Bilateral hilar prominence
> Interstitial infiltrations over bilateral lung fields and a consolidative lesion over L't upper lung field are noted. Infectious process may be compatible but concealed malignancy cannot be excluded, suggest clinical correlation and follow up.
> Degenerative change of thoracic and lumbar spine with marginal spur formation
> Compression fracture over T12, s/p vertebroplasty
> On CVC
Impression:
As above description.

特殊檢查

- 檢驗項目 **:Acid Fast Stain - Concentrated** 檢體**:Sputum** 報告日期 **:1090415**
  Result: NOT FOUND
  備註:

  檢驗項目 **:Acid Fast Stain - Concentrated** 檢體**:Sputum** 報告日期 **:1090414**
  Result: NOT FOUND
  備註:

  檢驗項目 **:Acid Fast Stain - Concentrated** 檢體**:Sputum** 報告日期 **:1090411**
  Result: NOT FOUND
  備註:

  檢驗項目 **:Mycobacteria Culture - solid & liquid** 檢體**:Sputum** 報告日期 **:1090407**
  1) Mycobaceterium tuberculosis (TB complex)
  2)

DRUG	SM	SM	INH	INH	RIF	EMB	EMB	PAS	K	EA	OFX
MCG/ML	2.0	6.0	0.2	1.0	1.0	5.0	10.0	8.0	6.0	5.0	2.0
RESULT											

  備註:

  檢驗項目 **:Acid Fast Stain - Concentrated** 檢體**:Sputum** 報告日期 **:1090323**
  Result: NOT FOUND
  備註:

  檢驗項目 **:Mycobacteria Culture - solid & liquid** 檢體**:Sputum** 報告日期 **:1090413**
  1) Mycobaceterium tuberculosis (TB complex)
  2)

DRUG	SM	SM	INH	INH	RIF	EMB	EMB	PAS	K	EA	OFX
MCG/ML	2.0	6.0	0.2	1.0	1.0	5.0	10.0	8.0	6.0	5.0	2.0
RESULT											

  備註:

  檢驗項目 **:Acid Fast Stain - Concentrated** 檢體**:Sputum** 報告日期 **:1090320**
  Result: NOT FOUND
  備註:

  檢驗項目 **:Acid Fast Stain - Concentrated** 檢體**:Sputum** 報告日期 **:1090320**
  Result: NOT FOUND
  備註:

病理報告

- 活體組織切片病理報告 手術日期：**109/03/25** 病理號：**S0908848**

診斷醫師:陳韻安 報告日期:109/04/01
Pathologic diagnosis:
1. Rectum, laparoscopic total mesorectal resection —- Adenocarcinoma, moderately differentiated. Tumor invaded to perirectal soft tissue and metastasized to perirectal lymph nodes (6/16) with multiple tumor deposits. The proximal cut end is free of tumor.
2. Cut end, distal, resection —- Mild hemorrhoid and unremarkable squamous mucosa.
3. IMA root, dissection —- Unremarkable lymph node (0/1) and fibrovasculoadipose tissue.

Ancillary study for diagnosis:
IHC stains for PMS2 and MSH6 are done on section B. Both markers are preserved.

Prognostic and predictive factor:
1. Tumor size: 4.5x3.5 cm.
2. Depth of invasion: Perirectal soft tissue
3. Histologic grade: G2: Moderately differentiated.
4. Angiolymphatic invasion: Present.
5. Perineural invasion: Present.
6. Tumor deposits (discontinuous extramural extension): Present, specify number of deposits: 7.
7. Macroscopic tumor perforation: Not identified.
8. Surgical Margins: a) Proximal Margin: Uninvolved by invasive carcinoma, distance of tumor from margin: 17.9 cm. b) Distal Margin: Uninvolved by invasive carcinoma, distance of tumor from margin: Cannot be assessed due to seperated specimen. c) Radial or Mesenteric Margin: Involved by invasive carcinoma (tumor present 0-1 mm from margin).
9. Lymph node status: Involved (6/17).
10. Extranodal involvement: Present.
11. Tumor regression grading S/P CCRT: No known presurgical therapy.
12. Type of polyp in which invasive carcinoma arose: Not identified.
13. Additional pathologic findings: Not identified.
14. Pathological TNM Stage: pT3N2a (According to the eighth edition, American Joint Committee on Cancer Staging Guidelines for Tumors).
15. TNM descriptors: Not applicable.

Gross description:
The specimen consists of 1) a segment of colon, 21.4 cm in length and 4.5 cm in proximal cut end circumference, 4.2 cm in distal circumference, and perirectal soft tissue. A gray tan firm annular elevated and centrally ulcerative tumor, 4.5x3.5 cm, is noted, very close to the distal cut end. On cross section, the tumor invades to the mesocolic soft tissue with 2 mm to the radial margin. The proximal cut end is free of tumor grossly. 2) distal cut end, a piece of tan mucosa-covered soft tissue, 2.1x1.8x0.8 cm. 3) IMA root, a piece of tan yellow soft tissue, 3.8x2.1x0.7 cm. Representative part for section: A) proximal cut end B-E) tumor and deep margin F) specimen 2 G) specimen 3 H-K) lymph nodes.

Note:
1. The interpretation of MMR panel and recommended studies are as below: No loss of nuclear expression of MMR proteins: low probability of microsatellite instability-high (MSI-H). Please note: There are exceptions to the above IHC interpretations. These results should not be considered in isolation, and clinical correlation with genetic counseling is recommended to assess the need for germline testing.
2. This case has been peer reviewed by two doctors.

#T-59600_2 #M-81403_2 2 1105 S0000F
#T-C4560_2 #M-81406_2 2 1105 000000

其它

- 檢驗項目 **:Anti-HBC IgG** 檢體**:Blood** 報告日期 **:1090317**
  CUTOFF VALUE : 1

- Result: * 0.036 coi * * Reactive *
  註：
  參考值： 1.≧1:Non-Reactive2. 1:Reactive

檢驗項目 :Anti-HBs 檢體:Blood 報告日期 :1090317
CUTOFF VALUE : 10
Result: * 175.4 IU/L * * Reactive *
註：
參考值： 10 IU/L: Non-Reactive≧10 IU/L: Reactive

檢驗項目 :Cholesterol & HDL-C 檢體:Blood 報告日期 :1090317
T.CHOL 194 (200, 961010 前 - 125-240)
HDL-C 59 mg/dl (M:>40, F:>50)
(961010前，皆 >40)
T.C./HDL-C 3.29 (5.0)

檢驗項目 :Clostridium difficile-toxin A & B 檢體:Stool 報告日期 :1090415
結果： Negative
OTHER
參考值： Negative

檢驗項目 :HbA1C 檢體:Blood 報告日期 :1090318
1 h P mg% 2 h p mg%
3 h P mg%
HbA1C: 17.1 GHb GLUCOSE,FASTING mg%
(4 - 6 %)
REMARK: 已複驗，若有需要，請重檢

檢驗項目 :LDL-C 檢體:Blood 報告日期 :1090317
LDL-C 114 mg/dl (130)

檢驗項目 :Bacterial Culture(Ordinary) 檢體:Drainage(penrose) 報告日期 :1090408
陰性結果：
陽 Pseudomonas aeruginosa 菌 H
性 Morganella morganii 落 H
結 數
果 目
Ampicillin Cefazolin ClindamycinClarithromyGentamicin Amikacin Chloromycin
(1) S S
(2) R>=64 S=1 S=2
(3)
(4)
Unasyn Penicillin GM(120ug) SMX+TMP Vancomycin Oxacillin Cefpirome
(1)
(2) R>=32 S=20
(3)
(4)
Carbapen(IPM)CefoperazonCefotaxime Ceftazidime Cefepime Ceftriaxone Flomoxef
(1) S S S
(2) R=4 S=8 S=0.12 S=0.12 S=0.25 I=16
(3)
(4)
MetronidazoleCiprofloxacMinocyclineTeicoplanin Tazocin CefmetazoneAztreonam
(1) S S
(2) S=0.25 S=4
(3)
(4)
Tigecycline Amox./Clav.Cefoxitin Levofloxa.Carbapen(MPM) Erythromycin
(1)
(2) S=8
(3)
(4)
Moxifloxacin Linezolid Ertapenem
(1)
(2) S=0.5
(3)
(4)

檢驗項目 :Bacterial Culture(Ordinary) 檢體:Drainage(Penrose) 報告日期 :1090408
陰性結果：
陽 Pseudomonas aeruginosa 菌 H
性 Morganella morganii 落 H
結 數
果 目
Ampicillin Cefazolin ClindamycinClarithromyGentamicin Amikacin Chloromycin
(1) S S
(2) R>=64 S=1 S=2
(3)
(4)
Unasyn Penicillin GM(120ug) SMX+TMP Vancomycin Oxacillin Cefpirome
(1)
(2) R>=32 S=20
(3)
(4)
Carbapen(IPM)CefoperazonCefotaxime Ceftazidime Cefepime Ceftriaxone Flomoxef
(1) S S S
(2) R=4 S=8 S=0.12 S=0.12 S=0.25 I=16
(3)
(4)
MetronidazoleCiprofloxacMinocyclineTeicoplanin Tazocin CefmetazoneAztreonam
(1) S S
(2) S=0.25 S=4
(3)
(4)
Tigecycline Amox./Clav.Cefoxitin Levofloxa.Carbapen(MPM) Erythromycin
(1)
(2) S=8
(3)
(4)
Moxifloxacin Linezolid Ertapenem
(1)
(2) S=0.5
(3)
(4)

出院時情況

- 3.改門診治療

出院指示

-

藥名	劑量	劑量單位	用法	頻次	數量	數量單位	現況	天數	單位
Norvasc tab 5mg	1	TAB	PO	QD	11	TAB	TH	11	天
Otilonium tab 40mg	1	TAB	PO	TID	33	TAB	TH	11	天
Smecta powder 3g	1	PKG	PO	QID	44	PKG	TH	11	天
Diovan tab 80mg	1	TAB	PO	QD	11	TAB	TH	11	天
$Miyarisan BM powd.	1	PACK	PO	TID	33	PACK	TH	11	天
Loperamide cap 2mg	1	CAP	PO	BID	22	CAP	TH	11	天
Allegra tab 60mg	1	TAB	PO	BID	22	TAB	TH	11	天
AKURIT-4 TAB	3	TAB	PO	QD	33	TAB	TH	11	天
Vit B6 tab 50mg	1	TAB	PO	QD	11	TAB	TH	11	天
C.B. oint 5gm		TUB	TOP	Q1HPRN	0	TUB	TH	1	瓶
50/850 Galvus Met	1	TAB	PO	BIDCC	22	TAB	TH	11	天
Livalo tab 2mg	1	TAB	PO	QN	11	TAB	TH	11	天
Toujeo inj 450U/1.5mL	15	U	SC	QN	1	U	TH	11	天
Ulstop tab 20mg	1	TAB	PO	BID	22	TAB	TH	11	天

出院計劃

- 門診日期
  - date on 2020/04/28
- 衛教 :1. DM and low lipid diet.
  2. Control blood pressure and blood sugar tightly.
- 下次預約掛號資訊
  1090421 (A_上午) 大腸直腸 2448診 113號
  1090421 (A_上午) 大腸直腸 2448診 115號
  1090428 (A_上午) 大腸直腸 2448診 90號
  1090428 (A_上午) 打針特診 3121診 52號
  1090428 (P_下午) 甲狀腺科 2310診 98號

主治醫師 1153B 王俊[illegible] / 住院醫師 2327J 王[illegible]

正式摘要(電子簽章：000000189139773)

W102 / W102

doctorRank:B
